# Efficacy and safety of nimotuzumab with neoadjuvant chemotherapy followed by concurrent chemoradiotherapy for locoregionally advanced nasopharyngeal carcinoma

**DOI:** 10.18632/oncotarget.17357

**Published:** 2017-04-21

**Authors:** Fangzheng Wang, Chuner Jiang, Zhimin Ye, Quanquan Sun, Tongxin Liu, Min Xu, Peng Wu, Kaiyuan Shi, Bin Long, Aizawa Rihito, Sakamoto Masoto, Zhenfu Fu

**Affiliations:** ^1^ Department of Radiation Oncology, Zhejiang Cancer Hospital, Zhejiang Hangzhou, People’s Republic of China; ^2^ Zhejiang Key Laboratory of Radiation Oncology, Zhejiang Hangzhou, People’s Republic of China; ^3^ Department of Breast Tumor Surgery, Zhejiang Cancer Hospital, Zhejiang Hangzhou, People’s Republic of China; ^4^ Department of Physics, Zhejiang Cancer Hospital, Zhejiang Hangzhou, People’s Republic of China; ^5^ Department of Pathology, Zhejiang Cancer Hospital, Zhejiang Hangzhou, People’s Republic of China; ^6^ Department of Ultrasonography, Zhejiang Cancer Hospital, Zhejiang Hangzhou, People’s Republic of, China; ^7^ Department of Nuclear Medicine, Zhejiang Cancer Hospital, Zhejiang Hangzhou, People’s Republic of China; ^8^ Department of Radiology, Japanese Red Cross Fukui Hospital, Fukui, Japan

**Keywords:** nasopharyngeal carcinoma, nimotuzumab, neoadjuvant chemotherapy, concurrent chemotherapy, intensity-modulated radiotherapy

## Abstract

We assessed the efficacy and safety of nimotuzumab plus neoadjuvant chemotherapy followed by concurrent chemoradiotherapy for Chinese patients with locoregionally advanced nasopharyngeal carcinoma. Clinical data from 210 nonmetastatic nasopharyngeal carcinoma patients diagnosed between May 2008 and April 2014 were retrospectively reviewed. All patients were initially treated with nimotuzumab plus neoadjuvant chemotherapy followed by concurrent chemoradiotherapy. Ninety-five patients received cisplatin-based adjuvant chemotherapy. The median follow-up duration was 48 months. Locoregional relapse and distant metastases occurred in 16 patients (16/210, 7.6%) and 18 patients (18/210, 8.6%), respectively. The 5-year local recurrence-free survival, regional recurrence-free survival, distant metastases-free survival, progression-free survival, and overall survival rates were 95.6%, 94.4%, 91.7%, 84.0%, and 88.7%, respectively. Univariate analysis revealed that concurrent chemotherapy regimens and clinical stage correlated with overall survival, and that adjuvant chemotherapy, N stage, clinical stage, and tumor response at the end of treatment were correlated with progression-free survival. In the multivariate analysis, concurrent chemotherapy regimens, clinical stage, and tumor response were important prognosticators. Grade 3/4 leukocytopenia was experienced by 24 patients (11.4%), and 6 patients (2.9%) developed mild liver damage during the period of neoadjuvant chemotherapy. Grade 3/4 acute mucositis was experienced by 13 patients (6.2%), and 12 patients (5.7%) experienced grade 3/4 leukocytopenia during the concurrent chemotherapy. The efficacy of nimotuzumab plus neoadjuvant chemotherapy followed by concurrent chemotherapy in locoregionally advanced nasopharyngeal carcinoma patients was encouraging and the toxicities were tolerable.

## INTRODUCTION

The incidence of nasopharyngeal carcinoma (NPC) is 15 to 50 cases per 100,000 annually in Southern China, Singapore, and Malaysia that vary with age, ethnicity, and geographical origin [1]. Radiotherapy (RT) is the standard treatment for NPC because of the anatomical location and the high radiosensitivity. Patients with locoregionally advanced (LA) NPC at diagnosis account for 60% to 70% of all NPC patients [2]. Intensity modulated radiation therapy (IMRT) has been used to improve locoregional control but provides little benefit for survival outcome and prevention of distant failure [3, 4]. According to meta-analyses of randomized studies, combination radiotherapy and chemotherapy reduces the risk of mortality by 18% and increases 5-year survival by 4% to 6% [5]. Concurrent chemoradiotherapy (CCRT) with or without adjuvant chemotherapy, which provides a benefit in overall survival, has become the standard treatment for LA NPC, although with acute toxicities [6-8]. A previous meta-analysis showed that compared with CCRT alone, addition of neoadjuvant chemotherapy (NAC) to CCRT reduces distant failure in LA NPC patients [9, 10], and another current meta-analysis confirmed that NAC followed by CCRT significantly improved progression-free survival (PFS) and overall survival (OS) [11]. However, the efficacy of the addition of NAC to CCRT in LA NPC patients remains controversial [12-14]. Considering these results, addition of NAC to CCRT has been a promising option for LA NPC patients in the era of IMRT. At present, the incidence of distant failure in LA NPC patients after combined treatment is more than 20% [15]. Therefore, new and effective regimens with tolerable toxicity for LA NPC are needed.

Overexpression of epidermal growth factor receptor (EGFR) is observed in many different cancers, including gliomas, sarcomas, and head and neck cancers [16]. Moreover, high EGFR expression is associated with poor prognosis [17, 18]. Several inhibitors of EGFR, such as cetuximab, panitumumab, erlotinib, and gefitinib, have shown favorable results in clinical trials [19, 20]. Cetuximab, the most commonly used anti-EGFR antibody, combined with radiotherapy (RT), has been shown to improve survival in patients with locoregionally advanced head and neck squamous cell carcinoma (LA HNSCC) [21]. In NPC, cetuximab with concurrent chemoradiotherapy is tolerable and has shown promising advantages for NPC prognosis [22]. However, the relatively high rate of mucositis and acne-like skin rash limit its clinical application [22, 23].

Nimotuzumab is a blocking anti-EGFR monoclonal antibody without intrinsic stimulating activity [24]. In the preclinical studies, nimotuzumab demonstrated antiproliferative, proapoptotic, and antiangiogenic activities [25], and nimotuzumab displayed a longer half-life and elevated area under the curve than cetuximab at the same dose level [26]. Nimotuzumab improves quality of life because it rarely causes severe dermatological toxicity, which is the most common adverse event resulting from cetuximab and panitumumab use [27].

Nimotuzumab has marketing approval for the treatment of LA NPC [28, 29]. However, the value of adding nimotuzumab to NAC followed by CCRT in LA NPC patients remains unclear. Therefore, we retrospectively investigated the safety and efficacy of nimotuzumab plus NAC followed by CCRT in LA NPC patients.

## RESULTS

### Patient characteristics and completion of treatment

Between May 2008 and April 2014, the clinical data of 210 newly diagnosed LA NPC patients, who were initially treated with nimotuzumab plus NAC followed by CCRT in the Department of Radiation Oncology, Zhejiang Cancer Hospital (Hangzhou, People’s Republic of China), were collected and retrospectively reviewed. Basic characteristics of patients are summarized in Table 1. All patients completed a full course of radical IMRT and received 1 to 4 cycles of NAC, ≥ 1 cycle of concurrent chemotherapy (CC), and 3 to 17 weeks of nimotuzumab (Table 2). Among these patients, 95 (45.2%) received adjuvant chemotherapy (AC).

**Table 1:** Basic characteristic of 210 LA NPC patients

Characteristic	*N* (%)
Gender	
Male	154 (73.3)
Female	56 (26.7)
Age (years)	
Range	13–72
Median	46
<50	131 (62.4)
≥ 50	35 (37.6)
WHO pathology	
Type I	9 (4.3)
Type II	3 (1.4)
Type III	198 (94.3)
ECOG performance status	
0	168 (80.0)
1	42 (20.0)
T stage *	
T1	7 (3.3)
T2	29 (13.8)
T3	96 (45.7)
T4	78 (37.2)
N stage *	
N0	21 (10.0)
N1	62 (29.5)
N2	108 (51.4)
N3	19 (9.1)
Clinical stage *	
III	117 (55.7)
IVa	75 (35.7)
IVb	18 (8.6)
Comorbidity	
No	154 (73.3)
Yes	56 (26.7)

Abbreviations: WHO: World Health Organization. ECOG: Eastern Cooperative Oncology Group.

*The 7th AJCC/UICC staging system.

**Table 2:** Completion of treatment in 210 patients with LA NPC

Treatment	*N* (%)
NAC regimens	
TPF	62 (29.5)
TP	64 (30.5)
GP	7 (3.3)
FP	73 (34.8)
Other	4 (1.9)
Cycle of NAC	
1	40 (19.0)
2	80 (38.1)
3–4	90 (42.9)
CC regimens	
Cisplatin	198 (94.3)
Non-cisplatin	12 (5.7)
Cycle of CC	
1	76 (36.2)
2	120 (57.1)
≥3	14 (6.7)
Period of CC	
Weekly	12 (5.7)
3 weeks	198 (94.3)
AC	
No	115 (54.8)
Yes	95 (45.2)
Fractional dose of h-R3	
100 mg	23 (11.0)
200 mg	187 (89.0)
Total dose of h-R3	
< 1200 mg	60 (28.6)
≥ 1200 mg	150 (71.4)
Cycle of h-R3	
< 6 weeks	39 (18.6)
≥ 6 weeks	171 (81.4)

Abbreviations: NAC: neoadjuvant chemotherapy. CC: concurrent chemotherapy. AC: adjuvant chemotherapy. h-R3: nimotuzumab.

### Disease response

At the end of treatment, complete remission (CR) and partial remission (PR) for lesions of the nasopharynx in 210 LA NPC patients accounted for 83.8% (176/210) and 16.2% (34/210), respectively. For 189 patients with neck metastatic lymph nodes, CR and PR rates of cervical lymph nodes were 88.9% (168/189) and 11.1% (21/189), respectively.

### Rates of local control and survival

**Figure 1:**
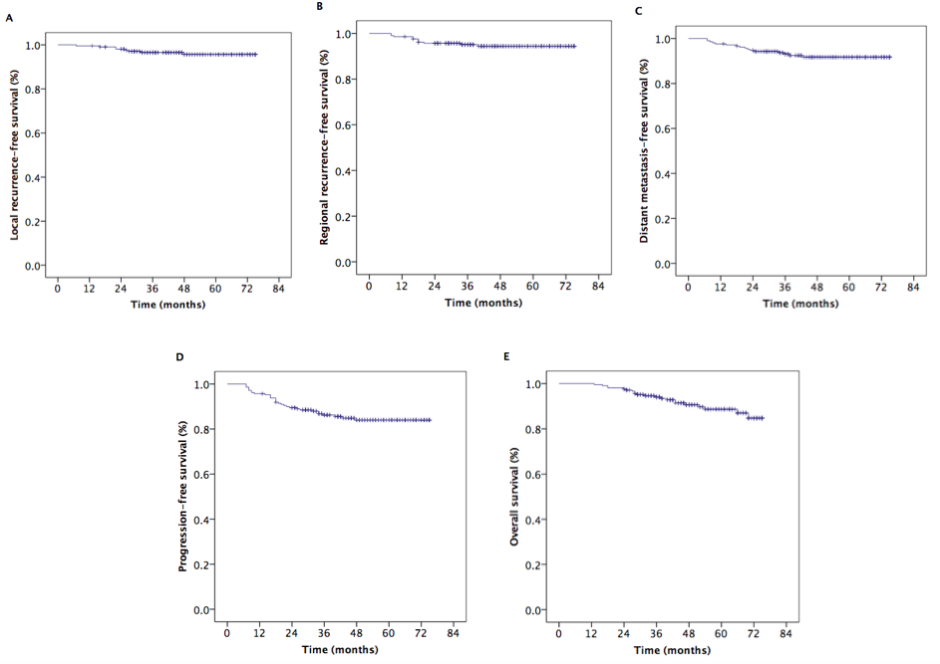
Kaplan–Meier estimates of the survival in patients with nasopharyngeal carcinoma **A.** Local recurrence-free survival, **B.** regional recurrence-free survival, **C.** distance metastasis-free survival, **D.** progression-free survival, and **E.** overall survival.

**Figure 2:**
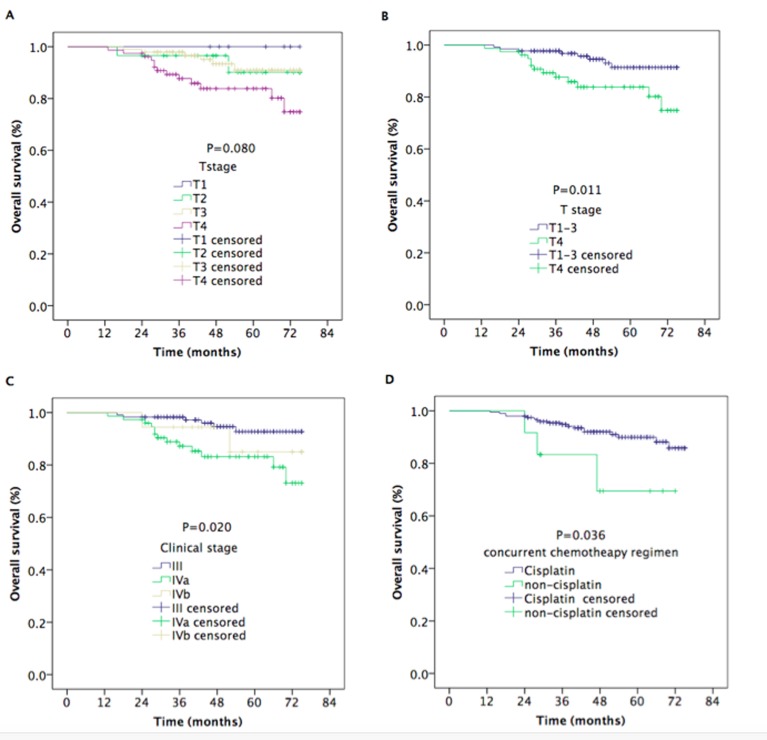
Kaplan–Meier estimates of the overall survival in nasopharyngeal carcinoma patients for different variable **A.** Overall survival for T stages, **B.** overall survival for stage T4 *vs*. T1-T3, **C.** overall survival for clinical stage, and **D.** overall survival for concurrent chemotherapy regimens.

**Figure 3:**
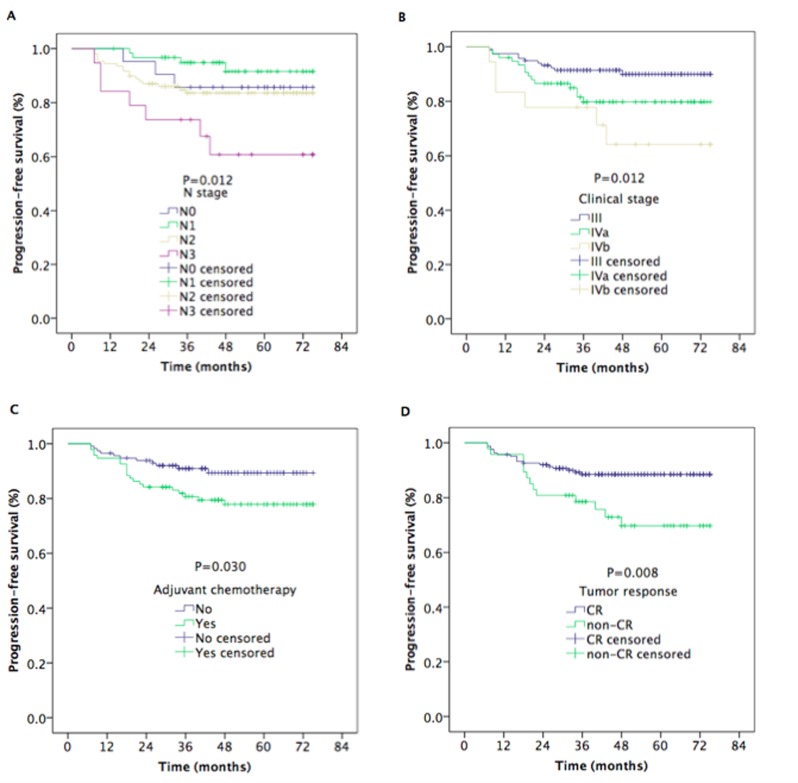
Kaplan–Meier estimates of the progression-free survival in nasopharyngeal carcinoma patients for univariate **A.** Progression-free survival for N stages, **B.** progression-free survival for clinical stages, **C.** progression-free survival of patients with or without AC, and **D.** progression-free survival for tumor response.

The median follow-up time was 48 months (range, 13-75 months). The estimated 5-year local recurrence-free survival (LRFS), regional recurrence-free survival (RRFS), distant metastasis-free survival (DMFS), progression-free survival (PFS), and overall survival (OS) rates were 95.6%, 94.4%, 91.7%, 84.0%, and 88.7%, respectively (Figure 1). The 5-year OS rates were 100%, 90.1%, 91.0%, and 83.8% for patients with stage T1, T2, T3, and T4 disease, respectively (*P* = 0.080) (Figure 2A). However, the patients with stage T4 disease had poorer OS rates than those with stage T1-T3 disease (83.8% *vs*. 91.4%, *P* = 0.011) (Figure 2B). The 5-year OS and PFS rates were 92.7%, 83.2%, and 85.0% and 89.9%, 79.8%, and 64.2% for patients with stage III, stage IVa, and stage IVb, respectively (*P* = 0.020) (Figure 2C), (*P* = 0.012) (Figure 3A). The 5-year OS rate of patients treated with a CC regimen of cisplatin was higher than that of non-cisplatin CC regimens (89.9% *vs*. 69.4%) (*P* = 0.036) (Figure 2D). The 5-year PFS rates were 90.2%, 89.9%, 89.2%, and 78.2% for patients with stage N0, N1, N2, and N3 disease, respectively (*P* = 0.012) (Figure 3B). The 5-year PFS rates for patients with AC *vs*. without AC and CR patients *vs*. non-CR patients were 89.4% *vs*. 77.9% (*P* = 0.030) (Figure 3C) and 88.5% *vs*. 69.8% (*P* = 0.008) (Figure 3D), respectively.

Treatment failure occurred in 31 patients by the last follow-up. Local relapse occurred in 7 patients, regional relapse occurred in 8 patients, and locoregional relapse occurred in 1 patient. Distant failure was experienced by15 patients. Patterns of treatment failure in NPC patients are listed in Table 3.

**Table 3:** Patterns of treatment failure in nasopharyngeal carcinoma patients

Sites	Number of patients (*n* = 31)
Local relapse only	7
Regional relapse only	8
Local and regional failure	1
Regional and distant failure	2
Distant failure only	13
Lung metastasis only	3
Bone metastasis only	2
Liver metastasis only	2
Lung, liver, bone and other	6

### Identification of prognostic factors

We evaluated the following potential prognostic factors: patient age, patient gender, clinical stage, adjusted tumor (T) and lymph node (N) stage, NAC, CCRT, AC, comorbidities, dose of nimotuzumab, and tumor response at the end of treatment. Univariate analysis revealed that clinical stage and CCRT regimens were significant prognostic factors for OS, and N stage, clinical stage, and tumor response at the end of treatment were significant prognostic factors for PFS (Table 4). In multivariate analysis, stage IV was a poorer prognostic factor for OS, PFS and DMFS, as were CC regimen of non-cisplatin for OS and DMFS and non-CR for OS and RRFS (Table 5).

**Table 4:** Univariate analysis of prognostic factors on OS and PFS in LA NPC patients

Characteristic	*N*	5-year OS (%)	*P*	5-year PFS (%)	*P*
Gender			0.319		0.058
Male	154	86.6		80.7	
Female	17	94.0		92.6	
Age (years)			0.124		0.870
<50	131	90.5		83.6	
≥ 50	79	85.4		84.8	
T stage *			0.080		0.361
T1	7	100.0		85.7	
T2	29	90.1		81.4	
T3	96	91.0		88.4	
T4	78	83.8		79.3	
N stage *			0.884		0.012
N0	21	90.2		85.7	
N1	62	89.9		91.6	
N2	108	89.2		83.6	
N3	19	78.2		60.8	
Clinical stage *			0.012		0.012
III	117	92.7		89.9	
IVA	75	83.2		79.8	
IVB	18	85.0		64.2	
Comorbidity			0.193		0.640
No	154	89.5		83.2	
Yes	56	86.3		86.4	
NAC regimens			0.257		0.462
TPF	62	89.9		79.3	
TP	64	90.9		89.8	
GP#	7	-		-	
FP	73	83.3		82.1	
Other	4	100.0			
Cycle of NAC			0.447		0.169
1	40	84.8		74.9	
2	80	89.3		85.2	
3–4	90	89.8		87.0	
Period of CC			0.992		0.781
Weekly	12	85.7		84.1	
3 weeks	198	88.9		83.3	
CC regimens			0.036		0.230
Cisplatin	198	89.9		84.6	
Non-cisplatin	12	69.4		75.0	
AC			0.369		0.030
No	115	91.6		89.4	
Yes	95	86.3		77.9	
Fractional dose of h-R3			0.966		0.896
100 mg	23	86.7		87.0	
200 mg	186	88.7		83.4	
Total dose of h-R3			0.976		0.459
< 1200 mg	60	83.6		85.2	
≥ 1200 mg	120	84.3		90.0	
Tumor response			0.270		0.008
CR	163	90.8		88.5	
Non-CR	47	81.4		69.8	

Abbreviations: NAC: neoadjuvant chemotherapy. CC: concurrent chemotherapy. AC: adjuvant chemotherapy. h-R3: nimotuzumab. CR: complete response.

*The 7th AJCC/UICC staging system. # Patients, who treated with GP, lived fewer than 5 years

**Table 5:** Multivariate analysis of prognostic factors in LA NPC patients

	Characteristic	HR	95% CI	*P*-value
OS	CC regimen of cisplatin *vs*. non-cisplatin	0.256	0.074–0.886	0.031
	III *vs*. IV*	0.291	0.113–0.754	0.011
PFS	III *vs*. IV*	0.438	0.213–0.899	0.024
	CR *vs*. non-CR	0.441	0.210–0.926	0.031
LRFS	—	—	—	—
RRFS	CR *vs*. non-CR	0.300	0.091–0.994	0.049
	With *vs*. without AC	0.222	0.047–1.043	0.057
DMFS	CC regimen of cisplatin *vs*. non-cisplatin	0.207	0.058–0.734	0.015
	III *vs*. IV*	0.166	0.047–0.583	0.005

Abbreviations: OS: overall survival. PFS: progression-free survival. LRFS: local recurrence-free survival. RRFS: regional recurrence-free survival. DMFS: distant metastasis-free survival. CC: concurrent chemotherapy. AC: adjuvant chemotherapy.

*The 7th AJCC/UICC staging system.

### Safety and toxicity

The most common treatment-related acute adverse events included hematologic and non- hematologic toxicity (Table 6). During the period of NAC, hematologic toxicity was reported as grade 3 and worse in severity in 45 patients (21.4%). Of these patients, 8 experienced neutropenic fever. The toxicities can be tolerated without delaying the chemotherapy and interrupting radiotherapy by GMSF treatment. The gastrointestinal toxicities were mild or moderate, and patients recovered rapidly with or without symptomatic medication. During the period of CCRT, the grade 3-4 hematologic toxicities and radiotherapy-related oral mucositis were reported in 14 patients (6.7%) and 9 patients (4.3%), respectively. Grade 3 dermatitis was observed in 5 patients within the RT field. No acneiform eruptions were found among these patients.

**Table 6:** Toxicity of nimotuzumab plus NAC followed by CCRT with or without AC

Adverse events	During the period of NAC					During the period of CCRT				
	0	1	2	3	4	0	1	2	3	4
White blood cell	63	57	45	35	10	131	36	29	9	5
Leukocytopenia	72	52	41	32	13	132	34	31	10	3
Anemia	180	22	6	2	0	109	55	39	7	0
Thrombocytopenia	184	17	6	3	0	147	38	23	2	0
Liver function	198	8	4	0	0	192	15	3	0	0
Renal function	203	7	0	0	0	206	3	1	0	0
Mucositis	175	27	8	0	0	44	84	73	7	2
Dermatitis	210	0	0	0	0	0	138	67	5	0
Diarrhea	197	9	3	1	0	202	6	2	0	0
Nausea/vomiting	157	29	17	5	2	172	25	12	1	0

Abbreviations: NAC: neoadjuvant chemotherapy. CCRT: concurrent chemoradiotherapy.

The most commonly observed late complication was xerostomia. However, the degree typically decreased over time. The degree of dry mouth in most patients was mild-to-moderate at the time of the last follow-up, and 123 patients did not complain of xerostomia. Finally, 79 patients developed either unilateral or bilateral hearing impairment, and 11 patients were found to have temporal lobe damage, which was diagnosed during follow-up based on magnetic resonance imaging.

## DISCUSSION

With further research on the molecular mechanism of tumorigenesis and tumor development, targeted molecular therapy in patients with NPC will become the research focus. Overexpression of EGFR was detected in 94% of patients with NPC [18]. Cetuximab is a common anti-EGFR monoclonal antibody drug. It has a good curative effect in the treatment of NPC, with a 2-year PFS of 86.5% to 89.3% and a 3-year OS of 90.9% [22], but severe oral mucositis and itchy acneiform rash limit its application in NPC. To minimize cetuximab-related toxicities, a novel EGFR-targeted agent without toxicities is warranted.

Nimotuzumab, a humanized immunoglobulin G1 (IgG1) isotype monoclonal antibody with a unique safety profile and low skin toxicity, has been approved for the treatment of non-NPC HNSCC [16, 30]. The advantage of the drug is that the affinity constant is lower than that of cetuximab, allowing for high tumor uptake and low normal-tissue uptake [31]. Nimotuzumab requires bivalent binding for stable attachment, which makes the agent selectively bind to tumors with moderate-to-high EGFR levels. When EGFR expression is low, as in tissue, cetuximab still has high binding ability because of its higher affinity constant [31]. Our experiment confirmed that nimotuzumab sensitizes nasopharyngeal carcinoma cell line CNE-2 *in vitro* to RT and can reduce cancer cell proliferation, induce cell apoptosis, and change cell cycle distribution [32]. All these effects indicate that nimotuzumab plus RT can be utilized in the design of the clinical trial of NPC.

To date, only four small-scale studies on adding nimotuzumab to RT or CCRT for NPC patients have been conducted. In a retrospective paired study by Li et al [33], the OS and PFS rates for the nimotuzumab/RT treatment group were lower than those for cisplatin/RT treatment group, but in the stage II or the older than 60 years subgroups, no significant differences were seen for OS and PFS. Zhai et al reported that the addition of nimotuzumab to IMRT showed promising locoregional control and survival outcomes for LA NPC patients [29]. Huang et al [34] and Liu et al [28] found that concurrent administration of nimotuzumab and CCRT yielded encouraging survival outcomes in LA NPC patients, with tolerable treatment-related toxicity. For the first two studies, because of the severe acute sequela of CCRT, nimotuzumab, as a preferred substrate for cisplatin, increased the quality of life in selected NPC patients, with similar treatment outcomes. However, in the last two studies, nimotuzumab added into the intensive modality of NAC followed by CCRT improved the survival of LA NPC patients but with normal-tissue damage. Those outcomes will be the direction of further research.

This study investigated the efficacy and safety of adding nimotuzumab to NAC followed by CCRT for LA NPC patients. The study showed promising clinical outcomes, with a 5-year LRFS of 95.6%, a 5-year RRFS of 94.4%, a 5-year DMFS of 91.7%, a 5-year PFS of 84.0%, and a 5-year OS of 88.7%. Univariate analysis revealed that clinical stage and CCRT regimens are significant prognostic factors for OS, and N stage, clinical stage, and tumor response at the end of treatment are significant prognostic factor for PFS. Multivariate analysis indicated that stage IV is a poorer prognostic factor for OS, PFS and DMFS, as stage N2 to N3 is for PFS and DMFS, a CCRT regimen of non-cisplatin is for OS and DMFS, and a non-CR is for OS and RRFS. Although 34.7% of patients experienced grade ≥3 hematologic toxicity and 12.0% experienced grade ≥3 radiotherapy-related oral mucositis, only 5 patients were observed with grade 3 dermatitis within the RT field. No acneiform eruptions were found among these patients.

We found that nimotuzumab plus NAC before CCRT with or without AC in the treatment of LA NPC patients is safe and effective. However, because of the retrospective nature of the study, our results should be regarded as preliminary.

## CONCLUSION

We observed that the administration of nimotuzumab with NAC followed by CCRT in LA NPC patients was tolerated and showed promising clinic outcomes. Further randomized, controlled, multicenter phase III clinical trials are needed to confirm the therapeutic gain.

## PATIENTS AND METHODS

### Pretreatment

The patients enrolled into this study were hospitalized from May 2008 to April 2014 in the Department of Radiation Oncology, Zhejiang Cancer Hospital. The retrospective study was approved by the medical ethics committee of Zhejiang Cancer Hospital. The eligible patients met the following criteria: (i) histologically proven LA NPC, (ii) Eastern Cooperative Oncology Group performance status ≤ 1, (iii) completion of radical IMRT, (iv) received nimotuzumab plus NAC before CCRT, and (v) no previous anti-cancer treatment.

Patients had pretreatment evaluations that included complete histories, physical examinations, hematology and biochemistry profiles, chest radiographs, sonography of the abdomen, bone scans, magnetic response images of the nasopharynx, and nasopharyngoscopies. All patients were staged per the 2010 American Joint Committee on Cancer staging system. Tumor histology was classified per the World Health Organization classification.

**Figure 4:**
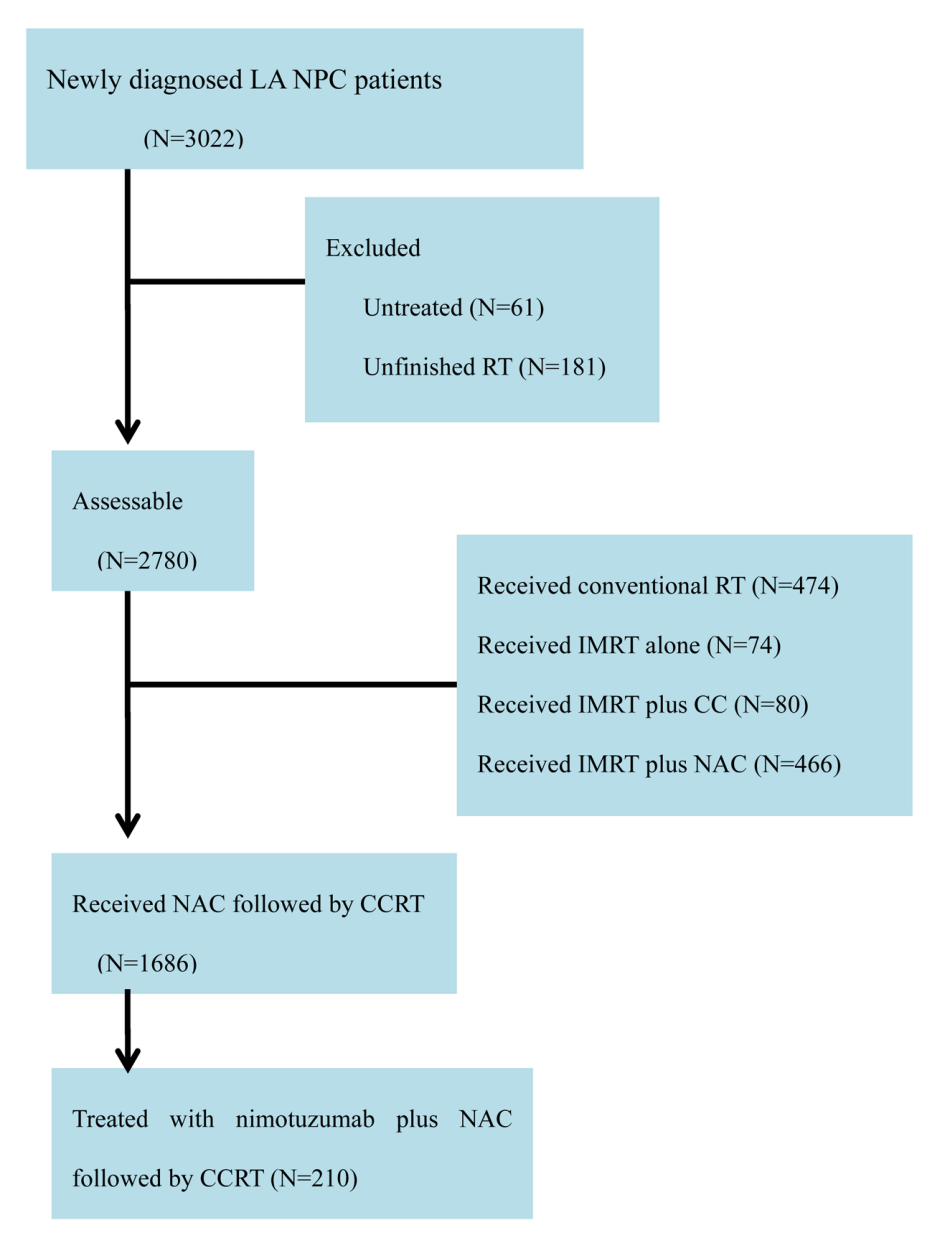
Flowchart of patients Abbreviations: NPC: Nasopharyngeal carcinoma; RT: Radiotherapy.

The flowchart of patients is shown in Figure 4. A total of 3022 newly diagnosed LA NPC patients were registered at Zhejiang Cancer Hospital. A total of 210 NPC patients treated with nimotuzumab plus NAC followed by CCRT were enrolled into this study. All patients received definitive IMRT with or without AC.

### Radiotherapy

All patients were immobilized in the supine position with thermoplastic masks. Computed tomography scans with intravenous contrast (2.5 mm slices from the head to 2 cm below the sternoclavicular joints) were performed for planning. All patients underwent radical IMRT with a simultaneous integrated boost technique that used 6-MV photons. The prescribed radiation doses were 69 Gy or 72 Gy to planning gross target volume (PGTV)nx, 66 Gy to 69 Gy to PGTVnd, 63 Gy to 66 Gy to planning target volume (PTV)nx, 60 Gy to 63 Gy to PTV1, and 51 Gy to 54 Gy to PTV2, delivered in 30 or 33 fractions. Radiation was delivered once daily, in five fractions per week, over 6 to 6.5 weeks for IMRT planning. The dose to organs at risk was limited based on the Radiation Therapy Oncology Group 0225 protocol.

### Target treatment

Nimotuzumab was administered concomitantly with induction chemotherapy and /or RT at a dose of 100 mg or 200 mg weekly, which was diluted in 250 mL of saline to obtain a 100-mg or 200-mg suspension and intravenously infused over 1 hour. All patients received 3 to 17 weeks of nimotuzumab during the treatment.

### Chemotherapy

All patients were given one to four cycles of platinum-based induction chemotherapy. The most common induction regimens included TPF (docetaxel 60 mg/m^2^/day on day 1, cisplatin 25 mg/m^2^/day on days 1 to 3, and 5-fluorouracil 500 mg/m^2^/day on days 1 to 3), TP (docetaxel 60 mg/m^2^/day on day 1, cisplatin 25 mg/m^2^/day on days 1 to 3), GP (gemcitabine 1,000 mg/m^2^/day on days 1 and 8, cisplatin 25 mg/m^2^/day on days 1 to 3), and FP (cisplatin 25 mg/m^2^/day on days 1 to 3, and 5-fluorouracil 500 mg/m^2^/day on days 1 to 3).

NPC patients underwent ≥1 cycle concurrent chemotherapy with cisplatin (80 mg /m^2^) for 3 days, and 95 patients received two to three courses of adjuvant chemotherapy with FP regimen 3 weeks after RT.

### Patient evaluation and follow-up

The assessment of tumor response was performed thrice after the completion of induction chemotherapy, at the end of IMRT, and 3 months after irradiation, which was based on MRI and nasopharynx fiberscope per the Response Evaluation Criteria for Solid Tumors. Systemic chemotherapy adverse effects were graded per the National Cancer Institute Common Toxicity Criteria (NCI CTCAE, Version 3.0), and RT-induced toxicities were scored per the Acute and Late Radiation Morbidity Scoring Criteria of the Radiation Therapy Oncology Group.

All the subjects underwent weekly examinations for treatment response and toxicities during RT. Patients were followed every 3 months in the first 2 years, every 6 months from the third to the fifth year, and then annually. Each follow-up included careful examination of the nasopharynx and neck nodes by an experienced doctor. MRI scan of the nasopharynx, nasopharynx fiberscope, chest computed tomography radiograph, and ultrasound of abdomen were performed 3 months after the completion of RT and every 6 to 12 months thereafter. Additional examinations were performed when indicated to evaluate local relapse or distant metastasis.

### Statistical analysis

Survival curves were generated by use of the Kaplan–Meier method. The curves were compared by use of log-rank tests. Multivariate analysis was performed by use of Cox regression models to identify significant prognostic factors. Hazard ratios (HRs) and 95% confidence intervals (CIs) were calculated for each prognostic factor. IBM SPSS Statistics Version 19.0 was used for all data analysis. A *P* < 0.05 was considered statistically significant. Survival time was calculated from the date of diagnosis to the most recent follow-up or to either the date of relapse (event-free, local recurrence-free, or distant metastasis-free) or death (overall survival). After recurrence or metastasis, patients were given salvage therapy as determined by their physicians.
